# Sophisticated roles of tumor microenvironment in resistance to immune checkpoint blockade therapy in hepatocellular carcinoma

**DOI:** 10.20517/cdr.2024.165

**Published:** 2025-02-26

**Authors:** Yi-Zhe Zhang, Yunshu Ma, Ensi Ma, Xizhi Chen, Yue Zhang, Baobing Yin, Jing Zhao

**Affiliations:** ^1^Hepatobiliary Surgery Center, Department of General Surgery, Huashan Hospital, Fudan University, Shanghai 200040, China.; ^2^Liver Transplantation Center, Department of General Surgery, Huashan Hospital, Fudan University, Shanghai 200040, China.; ^3^Institute of Organ Transplantation, Fudan University, Shanghai 200040, China.; ^4^The Second School of Clinical Medicine, Zhejiang Chinese Medical University, Hangzhou 310053, Zhejiang, China.; ^5^Department of Hepatobiliary surgery, National Regional Medical Center, Binhai Campus of the First Affiliated Hospital, Fujian Medical University, Fuzhou 350212, Fujian, China.; ^6^Cancer Metastasis Institute, Fudan University, Shanghai 201206, China.; ^#^Authors contributed equally.

**Keywords:** Hepatocellular carcinoma, immunotherapy, immune-checkpoint blockade, tumor microenvironment

## Abstract

Hepatocellular carcinoma (HCC) remains a serious threat to global health, with rising incidence and mortality rates. Therapeutic options for advanced HCC are quite limited, and the overall prognosis remains poor. Recent advancements in immunotherapy, particularly immune-checkpoint blockade (ICB) targeting anti-PD1/PD-L1 and anti-CTLA4, have facilitated a paradigm shift in cancer treatment, demonstrating substantial survival benefits across various cancer types, including HCC. However, only a subset of HCC patients exhibit a favorable response to ICB therapy, and its efficacy is often hindered by the development of resistance. There are many studies to explore the underlying mechanisms of ICB response. In this review, we compiled the latest progression in immunotherapies for HCC and systematically summarized the sophisticated mechanisms by which components of the tumor microenvironment (TME) regulate resistance to ICB therapy. Additionally, we also outlined some scientific rationale strategies to boost antitumor immunity and enhance the efficacy of ICB in HCC. These insights may serve as a roadmap for future research and help improve outcomes for HCC patients.

## INTRODUCTION

Hepatocellular carcinoma (HCC) is the third leading cause of cancer-related death and the incidence is prevalently increasing worldwide^[1]^. Chronic viral hepatitis associated with HBV and HCV infection, alcohol- and non-alcohol steatohepatitis, and long-term exposure to aflatoxin are the major risk factors for HCC development. Despite advances in surgical resection, locoregional therapies, and liver transplantation, only a small fraction of HCC patients at the early stage are eligible for curative treatments. A number of HCC cases have already been unresectable and at the advanced stage at the time of initial diagnosis. The prognosis of HCC is still dismal, with 5-year overall survival (OS) of less than 15%^[2,3]^. Over the past decade, traditional molecular targeted treatments like sorafenib and lenvatinib have been the standard first-line therapy for advanced HCC^[4,5]^. These treatments have improved the median OS to 11-14 months by inhibiting the aberrant activation of multiple tyrosine kinase receptors, including VEGFRs, FGFRs, and PDGFRs, which are crucial in tumor growth and progression^[4]^. Recently, immunotherapies, particularly immune-checkpoint blockade (ICB) with anti-PD1/PD-L1 and anti-CTLA4, have revolutionized cancer management, offering encouraging survival benefits superior to targeted treatment in a subset of HCCs^[6,7]^. Nonetheless, the majority of HCCs remain insensitive to monotherapy with ICB. The underpinning mechanisms and optimal strategies to address the resistance urgently need to be elucidated.

The tumor microenvironment (TME) plays a critical role in HCC development and therapeutic response^[8]^. The TME is a complex ecosystem composed of cancer-associated fibroblasts (CAFs), tumor-associated macrophages (TAMs), tumor-associated neutrophils (TANs), myeloid‐derived suppressor cells (MDSCs), regulatory T cells (Tregs), natural killer cells (NKs), T and B cells, endothelial cells (ECs), soluble factors, extracellular vesicles (EVs), and extracellular matrix (ECM)^[9-11]^.

Cancer cells orchestrate various components of the TME to foster a suppressive milieu that can abolish antitumor immunity, facilitate immune evasion, and mediate resistance to ICB treatment^[12]^. Nowadays, single-cell RNA sequencing (scRNA-seq) and spatial multi-omics have emerged as powerful tools for unraveling cancer pathogenesis^[13,14]^. These pioneering technologies can comprehensively profile the landscape of the TME, illustrate intratumoral heterogeneity, pinpoint distinct cell subtypes and structures, decipher complex cell-to-cell communication, and shed new light on the mechanisms of ICB resistance in HCC.

In this review, we have retrieved the latest progression in immunotherapy for HCC, systematically summarized the sophisticated mechanisms by which various components of the TME regulate response to ICB response, and detailed some critical cell subtypes and cell communication patterns involved in ICB resistance. Additionally, we outlined some potential strategies to enhance antitumor immunity and improve the efficacy of ICB in HCC.

## THE COMPLICATED ROLES OF THE TME IN ICB RESISTANCE OF HCC

The TME of HCC is characterized by a chronic inflammatory background and immunosuppressive phenotype. There exists a complex interplay of various cellular and non-cellular components that affect HCC progression and response to therapy.

### CAFs and ICB resistance in HCC

CAFs are the key components of the TME and exert pleiotropic functions to promote HCC development and therapeutic resistance. CAFs can facilitate cancer cells in acquiring malignant behaviors such as malignant proliferation, epithelial to mesenchymal transition (EMT), and cancer-stem-like properties, all of which significantly impair drug response. Additionally, CAFs can interact with various immune and ECs in the TME to remodel ECM, form immune barriers and establish an immunosuppressive milieu, thereby impairing antitumor immunity and ICB efficacy^[15-22]^.

#### CAFs cooperate with TAMs and form barriers to impede ICB efficacy

Recent research found that HCC patients who do not respond to anti-PD-1 treatment exhibit an immune barrier near the tumor boundary, composed of CAFs and SPP1^+^ macrophages, which blocks the infiltration of cytotoxic T cells into the tumor^[23]^. Secreted phosphoprotein 1 (SPP1), also known as osteopontin (OPN), plays a key role in this process. Targeting SPP1^+^ macrophages to reduce CAF enrichment effectively improves the efficacy of anti-PD-1 treatment in mice models^[23]^. Another investigation reported that POSTN^+^ CAFs can recruit SPP1^+^ macrophages to construct an immune barrier that suppresses effector T cell infiltration by activating the IL-6/STAT3 pathway and increasing SPP1^+^ expression. POSTN, or periostin, is a secreted ECM protein. HCC patients with high levels of both POSTN^+^ CAFs and SPP1^+^ macrophages frequently get less benefit from immunotherapy^[24]^. Additionally, POSTN^+^ CAFs have been identified as key components of the onco-fetal niche, interacting with FOLR2^+^ macrophages and PLVAP^+^ ECs. This niche is associated with cancer EMT, malignant proliferation, Treg enrichment, and insensitivity to immunotherapy in HCC^[25]^. FOLR2 is the folate receptor 2 on cell membranes, while plasmalemma vesicle associated protein (PLVAP) is a transmembrane glycoprotein mainly expressed in ECs^[25]^. FAP^+^ CAFs, which are mainly located around tumor borders, also play a role in the TME. DAB2^+^ and SPP1^+^ TAMs support the functions of FAP^+^ CAFs through TGF-β, PDGF, and ADM^[26]^. Fibroblast activation protein (FAP) is a type II transmembrane serine protease with bifunctional enzyme activity, while disabled 2 (DAB2) is a signaling and adenylate derivate-binding protein that plays multiple roles in cell growth, differentiation, migration, and signal transduction. The presence of FAP^+^ CAFs and DAB2^+^ TAMs at the tumor boundary may lead to a worse prognosis and poor response to immunotherapy in HCC^[26]^.

#### CAFs contribute to the immunosuppressive TME and resistance to ICB treatment

In addition to shaping the immune barrier, certain subtypes of CAFs, such as CD36^+^ CAFs and Endosialin^+^ CAFs, can infiltrate tumors to establish immunosuppressive TMEs and compromise immunotherapy. Mechanistically, hepatic stellate cell (HSC)-derived CD36^+^ CAFs uptake oxidized low-density lipoprotein (OxLDL) and induce lipid peroxidation, activating the p38/CEBPs axis, thereby driving migration inhibitory factor (MIF) expression. MIF released by CD36^+^ CAFs promotes the recruitment and expansion of MDSCs in the TME. The communication of CD36^+^ CAF and MDSCs promotes iNOS activity and MDSC secretion, facilitating Treg enrichment while reducing effector T cell infiltration, thus establishing an immunosuppressive microenvironment in HCC^[27]^. Moreover, NF-κB activation in MDSCs stimulates IL-6 secretion, which activates the STAT3 pathway and induces cancer-stem properties in HCC cells. These complex interactions between CD36^+^ CAFs and MDSCs contribute to HCC’s resistance to immunotherapy, but the CD36 inhibitor SSO (sulfo-N-succinimidyl oleate) significantly improves the efficacy of anti-PD-1 therapy^[27]^. Endosialin^+^ CAFs have also been shown to diminish CD8^+^ T cell recruitment within the TME, affecting immunotherapy response in HCC^[28]^. Endosialin, also known as CD248 or Tumor Endothelial Marker 1, decreases the phosphorated activation and nuclear translocation of STAT1 in CAFs exposed to IFN-γ, thus reducing CXCL9/10 expression and release, consequently decreasing the infiltration and function of CD8^+^ T cells. The combination of an endosialin antibody synergistically enhances the response to anti-PD-1 treatment^[28]^.

### TAMs and ICB resistance in HCC

TAMs are the most abundant immune cells within the TME and have significantly immunosuppressive roles to impede tumor-killing activity and counteract ICB efficacy in HCC.

#### HCC releases various factors to instigate TAMs to induce ICB resistance

HCC cells can activate multiple signaling pathways to drive the expression of various chemokines and cytokines, which in turn attract macrophages and promote their differentiation into tumor-promoting M2 phenotype^[29,30]^.

Tumor cell-intrinsic transcriptional factor ZFP64 is remarkably elevated in anti-PD-1 tolerant HCC cases, and ZFP64 can initiate CSF1 expression and secretion to induce M2 transformation and facilitate immune evasion^[31]^. PKCα is the upstream kinase to phosphorylate ZFP64 at S226 and promote K63-linked ubiquitination, which promotes ZEP64 entry into nuclear to drive CSF1 transcription. The protein kinase inhibitor Gö6976 or targeted drug Lenvatinib can conquer anti-PD1 resistance by inactivating the PKCa/ZFP64/CSF1 axis^[31]^. TGF-β-elevated SOX18 transactivates CXCL12 and PD-L1 expression in HCC cells to aggravate lung metastasis of HCC^[32]^. CXCL12 promotes the recruitment of TAMs and Tregs through the CXCR4 receptor; consequently, TAMs and Tregs release more TGF-β to form a feedback loop, and constantly activate the SOX18 axis to upregulate CXCL12 and PD-L1 and foster the suppressive milieu. TGF-βR1 inhibitor Vactosertib or CXCR4 inhibitor AMD3100 synergistically improves the efficacy of anti-PD-L1 to control metastasis and HCC progression^[32]^. RNase I upregulation is frequent in non-responders undergoing Nivolumab (anti-PD-1) treatment^[33]^. HCC-released RNase I can trigger M2 polarization by activating ALK signaling in macrophages. ALK inhibitor crizotinib combined with anti-PD1 enhances T cell infiltration and promotes the accumulation of memory-like T cells, which significantly retards tumor growth and provides long-term immunity to inhibit tumor progression^[33]^. The elevated CacyBP was found to bind with Myd88 and increase its protein stability by blocking Siah-1-mediated proteasomal degradation in HCC^[34]^. The interaction between CacyBP and Myd88 modulates HDAC1-induced H3K9ac and H3K27ac modifications on the CX3CL1 promoter and stimulates CX3CL1 expression and secretion, leading to TAM accumulation and the establishment of an immunosuppressive niche. Silencing CacyBP improves the efficacy of anti-PD-1 therapy in HCC^[34]^. HCC cell-derived PEG2 induces the transition of CX3CR1^+^ TAMs under immune stress, and these CX3CR1^+^ TAMs secrete IL-27, which promotes CD8^+^ T cell exhaustion and facilitates immune evasion^[35]^. The COX2 inhibitor, Celecoxib, which targets PEG2/CX3CR1^+^ TAMs, can reverse T cell exhaustion and strengthen anti-PD1 therapy^[35]^. HCC-derived M2BP induces chemotaxis of Mac2^+^ macrophages, leading to the formation of macrophage-coated tumor clusters (MCTCs), which create an immunosuppressive niche to trap cytotoxic T cells outside HCC^[36]^. Disrupting MCTC with GB1107 reshapes the suppressive TME and restores T cell abundance. Anti-PD-1 combined with GB1107 before MCTC formation results in enhanced efficacy in inhibiting HCC development^[36]^. Additionally, APOC1 depletion has been demonstrated to promote ferroptosis in HCC, which enhances immune activation, reverses M2 differentiation of TAMs, and improves the efficacy of anti-PD1 immunotherapy in HCC^[37]^.

#### EVs are involved in TAM-related ICB response

Golgi membrane protein 1 (GOLM1) aggravates HCC progression by releasing EVs to induce immunosuppression^[38]^. Mechanistically, GOLM1 promotes the deubiquitination of PD-L1, thereby increasing its protein stability by binding to CSN5. This interaction facilitates the packaging of PD-L1 into exosomes, which are then transported into TAMs^[38]^. The exosomal PD-L1 from HCC to TAMs induces M2 polarization and impedes the cytotoxic function of CD^+^ T cells. Zoledronic acid can enhance the effectiveness of anti-PD-L1 therapy by reducing PD-L1^+^ TAM infiltration and alleviating CD8^+^ T cell inhibition^[38]^. M2-derived EVs enable HCC to decrease MISP and promote the nuclear translocation of IQGAP1, which activates STAT3 phosphorylation and augments the expression of its downstream PD-L1, thereby modulating CD8^+^ T cell apoptosis and impairing their cytotoxic killing capacity^[39]^. Additionally, EV-loaded CD38 siRNA decreases the production of immunosuppressive metabolite adenosine and promotes the differentiation of TAMs toward the M1 phenotype, which represses HCC growth and metastasis while reversing tolerance to anti-PD-1/PD-L1 treatment^[40]^.

#### Heterogeneous subtypes of TAMs contribute to immunosuppression and ICB tolerance in HCC

TAMs are highly heterogeneous, with various subtypes influencing ICB outcomes. scRNA-seq landscape in responsive and non-responsive HCC patients treated with anti-PD-1 therapy reveals a significant accumulation of TREM2^+^ macrophages in non-responsive samples, while TCR^+^ macrophages exhibit tumor-killing capacity^[41]^. Anti-CSF1R treatment diminishes the infiltration of TREM2^+^ macrophages, thereby enhancing the therapeutic effects of anti-PD1^[41]^. Triggering receptor expressed on myeloid cells 2 (TREM2) is a transmembrane receptor primarily expressed on myeloid cells. Another study in HCC found that transarterial chemoembolization (TACE) increases the abundance of TREM2^+^ macrophages in the TME^[42]^. These TREM2^+^ macrophages reduce CXCL9 expression and secretion, impairing the recruitment of CD8^+^ T cells. Additionally, TREM2^+^ macrophages enhance the release of Gal-1, which promotes PD-L1 expression on endothelial vessel cells, thereby inhibiting CD8^+^ T cells. TREM2 deficiency or targeting Gal-1 improves the outcome of anti-PD-L1 treatment in HCC^[42]^. Under hypoxic conditions, HIF-1αupregulates TREM-1 and PD-L1 expression in macrophages. TREM-1^+^ macrophages activate the ERK/NF-κB signaling pathway to elevate CCL20 expression and secretion, leading to the recruitment of Tregs via the CCR6 receptor. The interplay between TREM-1^+^ macrophages and CCR6^+^ Tregs induces T cell apoptosis and dysfunction, thus weakening antitumor immunity^[43]^. Blocking TREM-1 with the specific inhibitor GF9 or anti-CCL20 restores the cytotoxic functions of CD8^+^ T cells and reverses resistance to anti-PD-L1 therapy^[43]^. Siglec-10hi TAMs, which share M2-like properties, are correlated with poor prognosis in HCC. Sialic acid-binding Ig-like lectin 10 (Siglec-10) is a transmembrane glycoprotein. Treatment with Siglec-10 Fc antibodies promotes CD8^+^ T cell activation, thus enhancing the efficacy of PD-1 inhibitor pembrolizumab in HCC^[44,45]^.

TAMs located in the adjacent margin of HCC also significantly impact antitumor immunity. Mucosal-associated invariant T (MAIT) cells, a major subtype of innate-like T cells, play a key role in modulating immunity and inflammation. CSF1R^+^ PD-L1^+^ TAMs in the adjacent HCC region interact with PD-1 on MAIT cells via cell-to-cell adhesion, sequestering MAITs away from the tumor core and impairing their cytotoxic capacity^[46]^. Anti-PD1/PD-L1 therapy can disrupt this interaction, increasing MAIT cell infiltration into the tumor and reinvigorating antitumor immunity in HCC^[46]^.

#### Subtypes of TAMs reinforce antitumor immunity and boost ICB efficacy

The abundance of NOD1^+^ TAMs has been associated with a favorable prognosis and sensitivity to anti-PD-1 treatments in HCC patients^[47]^. Nucleotide-binding oligomerization domain-containing protein 1 (NOD1) can decrease the expression of perilipin 5, which inhibits fatty acid oxidation and increases the accumulation of free fatty acids in TAMs. This metabolic reprogramming enables the costimulatory molecule OX40L to localize on the macrophage membrane, thereby activating CD8^+^ T cells^[47]^. Additionally, GSK3β deficiency in macrophages can inhibit M2 phenotype and thus HCC development. The GSK3β inhibitor, escitalopram, has been shown to synergistically improve the response to anti-PD1 treatment. Furthermore, upregulation of CD14^+^ GSK3β^+^ in peripheral blood mononuclear cells (PBMCs) has the potential to predict anti-PD1 insensitivity in HCC^[48]^.

### TANs and ICB sensitivity in HCC

TANs exhibit remarkable phenotypic plasticity, encompassing multiple distinct subtypes linked to inflammation, angiogenesis, and antigen presentation, as observed in pan-cancer neutrophil profiling analysis^[18,49,50]^. In HCC, TANs play critical roles in regulating ICB response.

#### HCC reprograms TANs to mediate ICB resistance

The accumulation of CD10^+^ ALPL^+^ neutrophils has been shown to lead to anti-PD-1 resistance in HCC^[51]^. HCC-derived NAMPT binds to the NTRK1 receptor on CD10^+^ ALPL^+^ neutrophils, inhibiting their maturation and activation. ALPL refers to Alkaline Phosphatase. These immature CD10^+^ ALPL^+^ neutrophils drive irreversible exhaustion of CD8^+^ T cells, which are characterized by high PD-1 and EOMES expression, through cell contact-dependent interactions, such as the SELL and SELPLG interaction^[51]^. The CD10^+^ ALPL^+^ neutrophil subtype presents a potential target for synergistic strategies to enhance anti-PD-1 effectiveness^[52]^. In HCC, the upregulation of CRKL prevents β-catenin degradation via the proteasome, promoting the expression of VEGF-A and CXCL1 and subsequently facilitating the recruitment and activation of PD-L1^+^ TANs^[52]^. The abundance of PD-L1^+^ TANs is associated with decreased T cell infiltration, low cytotoxic GZMB expression, and increased PD-1 and Tim3 engagement on CD8^+^ T cells^[52]^. Neutralizing antibodies targeting VEGF-A or CXCL1 can counteract CRKL-induced resistance to anti-PD-1 treatment^[52]^.

#### Neutrophil extracellular traps attenuate ICB response

Neutrophil extracellular traps (NETs) are web-like structures released by activated neutrophils, consisting of chromatin, neutrophil elastase, and myeloperoxidase. TAN-released NETs can promote immune evasion during HCC development^[53]^. Overexpression of GSK3a enhances the secretion of LRG1 from HCC cells, which facilitates neutrophil self-chemotaxis and NET formation by activating the NF-κB and STAT3 pathways. This process suppresses T cell infiltration and cytotoxic activity^[54]^. TAN-derived NETs can encapsulate tumor cells, shielding them from immune recognition by CD8^+^ T cells. GSK3a inhibitor (SB216763) can reverse immune evasion and improve anti-PD-1 efficacy^[54]^. Additionally, NETs-DNA can activate the Notch2/NF-κB signaling axis, increasing CD73 expression on HCC cells and promoting the infiltration of Tregs, thereby inducing immune escape^[53]^. Targeting NETs with agents like DNase I, in combination with anti-PD-1 therapy, can improve therapeutic outcomes^[53]^.

#### Specific subtypes of TANs enhance sensitivity to ICB

NASH-associated HCCs often exhibit a poor response to ICB therapy. A study found that CXCR2 antagonist targeting TANs can enhance the sensitivity to anti-PD-1 treatment in NASH-HCC. This effect is mediated by the reprogramming of TANs, activation of XCR1^+^ dendritic cells (DCs), and the enrichment of CD8^+^ T cells within tumors^[55]^. Furthermore, the study observed that TANs are increased in the TME and their phenotype switches from an immature state to a progenitor-like neutrophil state. These reshaped TANs directly contact with CD8^+^ T cells in a niche enriched with cytotoxic GZMB^[55]^. A novel subtype of TANs with antigen-presenting properties has recently been identified across various cancers and is correlated with a favorable prognosis^[49]^. Mechanistically, the activation of leucine metabolism induces epigenetic regulation of histone H3K27ac, which primes the generation of HLA-DR^+^ TANs. These HLA-DR^+^ TANs can trigger both (neo)antigen-specific and antigen-independent T cell responses. Neutrophil delivery or a leucine-enriched diet can improve anti-PD-1 therapy in multiple mouse cancer models, including HCC^[49]^.

### MDSCs and ICB resistance

MDSCs contribute to the immunosuppressive TME by releasing inhibitory molecules such as TGF-β, iNOS, IL-6, Arg1, and IL-10, thereby impairing the tumor-killing capacities of effector T cells and NK cells. MDSCs exert crucial roles in HCC resistance to ICB^[21,27,39,56-58]^.

#### Aberrant activation of epigenetic and transcriptional programs promotes MDSC infiltration and ICB tolerance

Epigenetic modifications, including RNA m6A methylation, N4-acetylcytidine (ac4C), and histone acetylation or methylation, significantly impact gene expression and cancer immunology^[59]^. In NASH-HCC, YTHDF1, an important m6A reader, binds to m6A-modified EZH2 RNA, promoting its translation. Subsequently, EZH2 initiates the expression and release of IL-6, which attracts MDSC infiltration and suppresses the cytotoxic functions of CD8^+^ T cells^[60]^. Depletion of YTHDF1 effectively reduces MDSC accumulation, restores CD8^+^ T cell function, and enhances anti-PD-1 efficacy^[60]^. In myeloid cells, reduced ac4C modification promotes MDSC infiltration while decreasing CD8^+^ T cell enrichment and activity, ultimately impairing immunotherapy efficacy^[59]^. In fibrotic HCC, activated HSCs mediate enhancer reprogramming of monocytic MDSCs by activating the monocyte-intrinsic P38/MAPK signaling pathway, which induces H3K27ac in C/EBPβ and S100A8/9/12 enhancers^[61]^. These M-MDSCs impair CD8^+^ T cell function and weaken immune surveillance^[61]^. BET inhibitors, which target epigenetic regulators, can suppress M-MDSC-mediated immunosuppression, thereby enhancing the efficacy of PD-L1 blockade^[61]^.

Additionally, HCC can upregulate the transcriptional factor PPARγ to drive VEGF-A release, further recruiting MDSCs to foster the immunosuppressive TME^[62]^. The PPARγ antagonist can avert resistance to anti-PD-L1 treatment^[62]^. LAPTM4B, a lysosome-associated membrane protein, facilitates MDSC migration and accumulation by increasing CXCL8 (IL-8) secretion^[63]^.

#### Ferroptosis correlates with MDSC recruitment to mediate ICB resistance

Ferroptosis is a distinct form of programmed cell death driven by iron accumulation and lipid peroxidation, which can induce a complex immune response. GPX4-dependent ferroptosis in hepatocytes can promote CXCL-10-induced CD8^+^ T cell infiltration, thereby inhibiting HCC progression^[64]^. However, this tumor-suppressive effect is counteracted by PD-L1 elevation and HMGB1-mediated MDSC infiltration. The ferroptosis inducer withaferin A and the CXCL10/CXCR2 axis inhibitor SB225002 can significantly restrain HCC metastasis and improve the prognosis of anti-PD-1 treatment^[64]^. MerTK activation contributes to resistance to anti-PD-1/PD-L1 therapy by inhibiting ferroptosis and promoting MDSC recruitment^[65]^. Inhibition of MerTK with Sitravatinib can reactivate ferroptosis, reduce MDSC infiltration, and enhance the antitumor response of CD8^+^ T cells, thereby overcoming resistance to ICB therapy^[65]^.

#### Metabolic disorders lead to MDSC expansion to impair ICB response

Squalene epoxidase (SQLE), a rate-limiting enzyme in cholesterol biosynthesis, drives cholesterol accumulation, which not only impairs the effector function of tumor-infiltrating CD8^+^ T cells but also promotes MDSC activation. Arg1^+^ and iNOS^+^ MDSCs secrete inhibitory factors that suppress T cell activity^[66]^. SQLE inhibitors, such as terbinafine, can reduce the immunosuppressive activity of MDSC, restore CD8^+^ T cell function, and improve the efficacy of PD-1 inhibitors in HCC^[66]^. Elevated glycolysis in HCC cells leads to lactate accumulation, which promotes MDSC infiltration and function within the TME. The upregulation of STAT3 and IDO1 further drives MDSC accumulation^[67]^. Glycolysis inhibitors, such as dichloroacetate (DCA), reduce lactate production, inhibit STAT3 activation and IDO1 expression, and ultimately decrease MDSC infiltration^[67]^.

#### MDSC accumulation induced by locoregional therapies contributes to insensitivity to ICB treatment

Locoregional therapies, such as TACE with 5-fluorouracil (5-FU), can induce MDSC accumulation in HCC, particularly polymorphonuclear MDSCs (PMN-MDSCs), thereby weakening the antitumor effects of CD8^+^ T cells and NK cells^[68]^. Depletion of MDSCs with anti-Ly6G restores the efficacy of anti-PD-L1, significantly improving ICB outcomes^[68]^. Incomplete radiofrequency ablation (iRFA) creates a hypoxic microenvironment in residual tumors, promoting the upregulation of HIF-1α and accelerating MDSC recruitment and accumulation^[69]^. The combination of melatonin significantly enhances the efficacy of anti-PD-L1 therapy^[69]^.

### Targeting Tregs to enhance sensitivity to ICB therapy in HCC

Tregs play a vital role in maintaining immune homeostasis, but also serve as main impediments to antitumor immunity. The abundance of Tregs is notably correlated with HCC progression and ICB response^[21,31,62]^.

The hypoxia TME promotes the CCL20- and CXCL-5-associated recruitment of Tregs and cDC2 cells in HCC, and the interaction between Tregs and cDC2 cells inhibits antigen-presenting HLA-DR expression on cDC2 cells and impairs antitumor immunity in HCC^[70]^. HCC-released GDF15 stimulates the generation of peripheral inducible Tregs (iTregs) and promotes the repressive function of natural Tregs (nTregs)^[71]^. Mechanistically, GDF15 binds to its receptor CD48 on T cells, which downregulates the expression of E3 ligase STUB1, leading to increased stability of FOXP3 in Tregs. Targeting GDF15 can restore antitumor immunity^[71]^.

Furthermore, the upregulation of SOX12 in HCC promotes Treg infiltration by transcriptionally increasing the expression and secretion of CCL22, which engages with the CCR4 receptor on Tregs to suppress CD8^+^ T cells^[72]^. TGF-β1 /TGF-β1R and Smad2/3/4 are upstream signaling for the upregulation of SOX12. Combination therapy with the CCR4 inhibitor or TGF-β1R inhibitor galunisertib can sensitize HCC to anti-PD-L1 treatment^[72]^. CCR4^+^ Tregs have been demonstrated as the main Treg subtype with PD1^+^ TCF1^+^ stem-like properties in HBV-associated HCC^[56]^. CCR4^+^ Tregs secrete IL-10 and IL-35, which dampen CD8^+^ T cell function. The neutralizing pseudo-receptor N-CCR4-Fc, which binds to the ligand CCL22, selectively blocks the intratumoral infiltration of Tregs and enhances sensitivity to anti-PD1 therapy^[56]^.

### HCC induces NK cell dysfunction to modulate ICB efficacy

NK cells, as essential components of the innate immune system, can directly eliminate cancer cells without antigen sensitization or MHC-I molecules. Additionally, NK cells play a key role in orchestrating immune responses by releasing various cytokines and chemokines. They also mediate antibody-dependent cellular cytotoxicity (ADCC), a crucial mechanism that enhances the effectiveness of ICB efficacy^[73]^. However, HCC and the malignant TME may employ multiple mechanisms to induce NK cell inactivation, thereby aggravating cancer progression and reducing the efficacy of ICB therapy.

HCC-derived exosomes are enriched with circUHRF1, which can be transferred into NK cells, inducing their exhaustion^[74]^. Mechanistically, circUHRF1 can act as a sponge to degrade miR-499c-5p, thus increasing the expression of its target, TIM-3, on NK cells, which leads to NK cell dysfunction and anti-PD1 resistance^[74]^. Recent studies have reported that the sialic acid-binding receptor Siglec-9 is upregulated on tumor-infiltrating NK cells and correlates with poor prognosis in HCC patients^[75]^. The binding of Siglec-9 to its ligand activates inhibitory signaling pathways, suppressing cytolytic activation and reducing NK cell secretion. Targeting Siglec-9 with the inhibitor MTX-3937 can restore NK cell tumor-killing function and enhance the efficacy of anti-PD-1 therapy^[75]^. Furthermore, the abundance of TGF-β in the HCC milieu has been found to stimulate the transition of NK cells into ILC1 cells, thereby attenuating the tumor-killing capacity of NK cells and facilitating immune escape^[76]^.

### CD8^+^ T cells and ICB response in HCC

CD8^+^ T cells are the most important component of antitumor immunity. However, tumors have developed a variety of mechanisms to compromise the tumor-eliminating capacities of CD8^+^ T cells via inhibiting their chemotaxis into tumors, inducing exhaustion, and impairing their cytotoxic function. The activity and status of CD8^+^ T cells play a decisive role in ICB response in HCC.

#### PD-1/PD-L1 axis exhausts T cell function and affects ICB response

The PD-1/PD-L axis is a well-known immune checkpoint pathway to induce CD8^+^ T cell exhaustion and immune evasion. Multiple mechanisms regulate this axis and ICB resistance in HCC.

HCC cells can encapsulate circCCAR1 in exosomes and release them into the TME. Upon uptake by CD8^+^ T cells, circCCAR1 directly binds to PD-1 protein, preventing its ubiquitination and degradation, thereby prolonging PD-1 stability and expression. This regulation induces CD8^+^ T cell exhaustion, contributing to resistance to ICB^[77]^. mTORC1 bidirectionally regulates PD-L1 expression in HCC, depending on TP53 status^[78]^. Specifically, HCC cells with TP53 loss-of-function mutations upregulate PD-L1 expression through activation of the mTORC1 pathway, whereas HCC with wild-type TP53 increases PD-L1 expression via mTORC1 inhibition. The combination of mTOR inhibitors can improve the efficacy of anti-PD-L1 in HCC with wild-type TP53^[78]^. Gasdermin D (GSDMD) also contributes to increased PD-L1 expression in HCC by activating the Ca^2+^/HDACs/STAT1 signaling pathway and inducing K^+^ efflux, which inhibits the cGAS pathway and reduces type I interferon production, weakening ICB efficacy^[79]^. Combining the GSDMD inhibitor DMF with ani-PD-1 improves CD8^+^ T cell infiltration and activity, thus enhancing ICB efficacy and reducing resistance^[79]^. SLAMF7 deficiency can lead to upregulation of PD-1 on CD8^+^ T cells, further impairing their function and making HCC resistant to ICB^[80]^. Co-administration of CCL2/CCR2 inhibitors with PD-1 antibodies can inhibit tumor growth and improve ICB outcomes^[80]^.

PD-L1 is also extensively expressed on immune cells within the TME. Compared to microwave ablation (MWA), cryoablation (CRA) induces higher PD-L1 expression, particularly in CD11b^+^ myeloid cells, creating an immunosuppressive microenvironment that limits CD8^+^ T cell activity and leads to ICB resistance^[73]^. Combining CRA with PD-L1 antibodies enhances CD8^+^ T cell responses, reduces immunosuppressive cells, and overcomes resistance, making it a potential treatment for advanced or unresectable HCC^[73]^. IgA^+^ monocytes and macrophages, with high PD-L1 expression, directly inhibit CD8^+^ T cell function, leading to T cell exhaustion and reduced cytotoxicity. This phenomenon represents a key factor in HCC resistance^[81]^. Blocking the IgA signaling pathway can reduce IgA^+^ PD-L1-high cells, restore T cell function, and overcome resistance^[81]^.

#### Metabolic reprogramming of HCC impairs T cell function and ICB efficacy

Metabolic reprogramming is a significant hallmark of cancer development. The aberrant accumulation of metabolites in the TME has a profound impact on the antitumor function of T cells and ICB response in HCC.

Glucometabolic reprogramming in HCC can impair the cytotoxic function of T cells. The serine- and arginine-rich splicing factor SRSF10 promotes glycolysis in HCC by stabilizing MYB RNA, which in turn increases the expression of glycolysis enzymes GLUT1, HK1 and LDHA, thus leading to lactate accumulation in the TME and histone lactylation in macrophages, and consequentially a reduced infiltration of CD8^+^ T cells^[82]^. Targeting SRSF10 with the pharmacological inhibitor 1C8 remarkably enhances the efficacy of anti-PD-1^[82]^. Furthermore, the downregulation of glutaryl-CoA dehydrogenase (GCDH) increases the crotonylation modification of glucometabolic proteins PGD, TKT, and ALDOC, thereby limiting glycolysis, reducing lactate production, and inducing HCC cell senescence^[83]^. HCC patients with low GCDH expression are sensitive to anti-PD-1 treatment^[83]^.

Abnormal cholesterol metabolism may deprive the antitumoral activity of T cells. Previous studies have demonstrated that HCC cells activate the SULT2B1 enzyme to synthesize cholesterol sulfate (CS), which directly inhibits DOCK2 enzyme activity. This disrupts mitochondrial function and induces CD8^+^ T cell exhaustion^[84,85]^. Inhibiting SULT2B1 or reducing CS production with tolazamide can improve the efficacy of anti-PD-1 therapy^[84]^. In NAFLD-HCC, overexpression of METTL3 promotes mRNA m^6^A modification and enhances SCAP translation, which activates cholesterol synthesis pathways^[86]^. The accumulation of cholesterol and cholesteryl esters in the TME diminishes the infiltration of INF-γ^+^ and GZMB^+^ CD8^+^ T cells, impairing their function. Targeting METTL3, particularly in combination with anti-PD-1, has shown a synergistic effect in blocking tumor progression^[86]^.

Aberrant tryptophan metabolism in HCC results in kynurenine accumulation, which mediates CD8^+^ T cell exhaustion and compromises ICB sensitivity. The transcriptional factor ZNF207 stimulates IDO1 expression, increasing kynurenine levels in the TME, thereby weakening the tumor-killing capacity of CD8^+^ T cells^[87]^. The silence of ZNF207 potentiates the response to anti-PD-1 in HCC mice models^[87]^. High expression of the immunoglobulin RBPJ is associated with diminished cytotoxicity in CD8^+^ T cells^[88]^. Inhibiting RBPJ with RIN1 blocks the mTOR pathway, reduces L-kynurenine synthesis, and decreases kynurenine levels in HCC cells, thus alleviating T cell exhaustion^[88]^. RIN1 treatment also enhances the effectiveness of anti-PD1 and PD-L1 therapies^[88]^.

#### Dysregulation of T cells impairs antitumor immunity and ICB efficacy

NASH is commonly unsensitive to ICB treatment, despite abundant CD8^+^ T cell infiltration within the TME^[89]^. Further investigations uncovered that NASH impairs the mobility and mitochondrial fitness of CD8^+^ T cells by inducing mitochondrial depolarization and mass reduction, while also decreasing glycolysis in intratumoral CD8^+^ T cells. Metformin can enhance the efficacy of anti-PD-1, as well as the combination of anti-PD-L1 and VEGFR2 inhibitors^[89]^. CTNNB1 mutations can transcriptionally activate MMP9 expression, increasing its secretion from HCC cells^[90]^. MMP9 cleaves the SSH1 protein on the surface of CD8^+^ T cells, inhibiting the activation of CXCR3-mediated GPCR signaling, thereby impairing the migration and antitumoral function of CD8^+^ T cells. Blocking MMP9 can alleviate resistance to ani-PD-1 therapy^[90]^.

Refinement of T cell function is essential to boost antitumor immunity. IFNα therapy exerts a remarkable synergistic effect when combined with anti-PD-1 in HCC patients, associated with the enrichment of cytotoxic CD27^+^ CD8^+^ T cells^[91]^. Mechanistically, IFNα treatment limits glucose metabolism in HCC by inhibiting the FosB/HIF axis, thereby fostering a high-glucose TME. This environment provides adequate glucose for CD8^+^ T cells, activating the mTOR/FOXM1signaling pathway and promoting the expression of the costimulatory molecule CD27 on infiltrating CD8^+^ T cells^[91]^. A downregulation of TPX4 was observed in HCC-infiltrating CD8^+^ T cells, significantly limiting their antitumor activity^[92]^. TPX2 can activate the NF-κB signaling pathway to drive CXCR5 expression on CD8^+^ T cells, thus enhancing the recruitment and cytotoxic function of CD8^+^ T cells in HCC. Overexpression of TPX2 can synergistically improve the efficacy of anti-PD-1 in HCC^[92]^.

#### Targeting HCC restores T cell immunity and enhances ICB sensitivity

The antigen presentation process is critical to boost antitumor immunity. However, cancer cells frequently downregulate MHC-I expression and show poor antigen-presenting capacity, thereby facilitating immune evasion. A recent study revealed that inhibiting FASN can protect MHC-I from lysosomal degradation and increase MHC-I levels on the surface of HCC cells, thus enhancing CD8^+^ T cell cytotoxicity^[93]^. FASN inhibitors such as orlistat or TVB-2640 robustly enhance sensitivity to anti-PD-L1 treatment in HCC^[93]^. The upregulation of PRMT2 is notably linked to resistance to ICB treatment in HCC patients^[94]^. Targeting PRMT3 in HCC can effectively restore the antitumor response, as PRMT3 inhibition disrupts mitochondrial integrity and induces mtDNA leakage. The released mtDNAs activate the cGAS/STING pathway, triggering the tumor-suppressive function of CD8^+^ T cells. Blocking PRMT3 functions can overcome resistance to anti-PD-1 in HCC^[94]^. Additionally, the combination of anlotinib and anti-PD-1 shows a strong synergistic effect in preclinical HCC models^[95]^. Anlotinib treatment inhibits the VEGFR2/AKT/HIF-1α signaling axis, reducing TRFC expression and increasing CXCL14 production in HCC, thereby mediating CD8^+^ T cells chemotaxis and enhancing the efficacy of anti-PD-1 in HCC^[95]^.

### ECs and ICB resistance in HCC

HCC is characterized by dysregulated angiogenesis. The overexpression of pro-angiogenic factors such as VEGF, Ang2, and TGF-β stimulates vascular ECs to form capillaries with abnormal structure and function^[96,97]^. The combination of anti-PD-L1 and anti-VEGF has been approved as the first-line systemic therapy for unresectable HCC^[98]^. This synergistic effect likely contributes to vascular normalization, thus facilitating drug delivery, improving the hypoxia microenvironment, and promoting the infiltration of effector cytotoxic lymphocytes into the tumor^[98,99]^.

Beyond angiogenesis, vascular ECs can also contribute to the formation of an immunosuppressive milieu. For instance, hypoxia-driven DGKG expression on vascular ECs induces Treg differentiation by activating a positive feedback loop in the TGF-β signaling pathway^[100]^. Mechanistically, DGKG interacts with USP16 to mediate the deubiquitination of ZEB2, enhancing its protein stability, thus elevating TGF-β release and Treg accumulation. Targeting DGKG inhibits both tumor angiogenesis and Treg differentiation, thereby enhancing the efficacy of combined anti-PD-1 and anti-VEGFR2 therapies^[101]^. In addition to vascular ECs, liver sinusoidal endothelial cells (LSECs) play a critical role in forming the specialized vascular bed structure that ensures adequate blood supply to the hepatic parenchyma, supporting normal liver functions such as detoxification, metabolism, and immune responses. However, under pathological stimuli, LSECs often undergo capillarization and activate HSCs, which promote fibrosis and HCC development. Nano delivery of simvastatin can prevent the crosstalk between LSECs and HSCs, alleviate LSEC capillarization, and upregulate CXCL16 expression in LSECs to recruit NKT cells^[100]^. The combination of simvastatin and anti-PD-L1 boosts antitumor immunity and improves therapeutic outcomes in HCC^[100]^.

**Figure 1:**
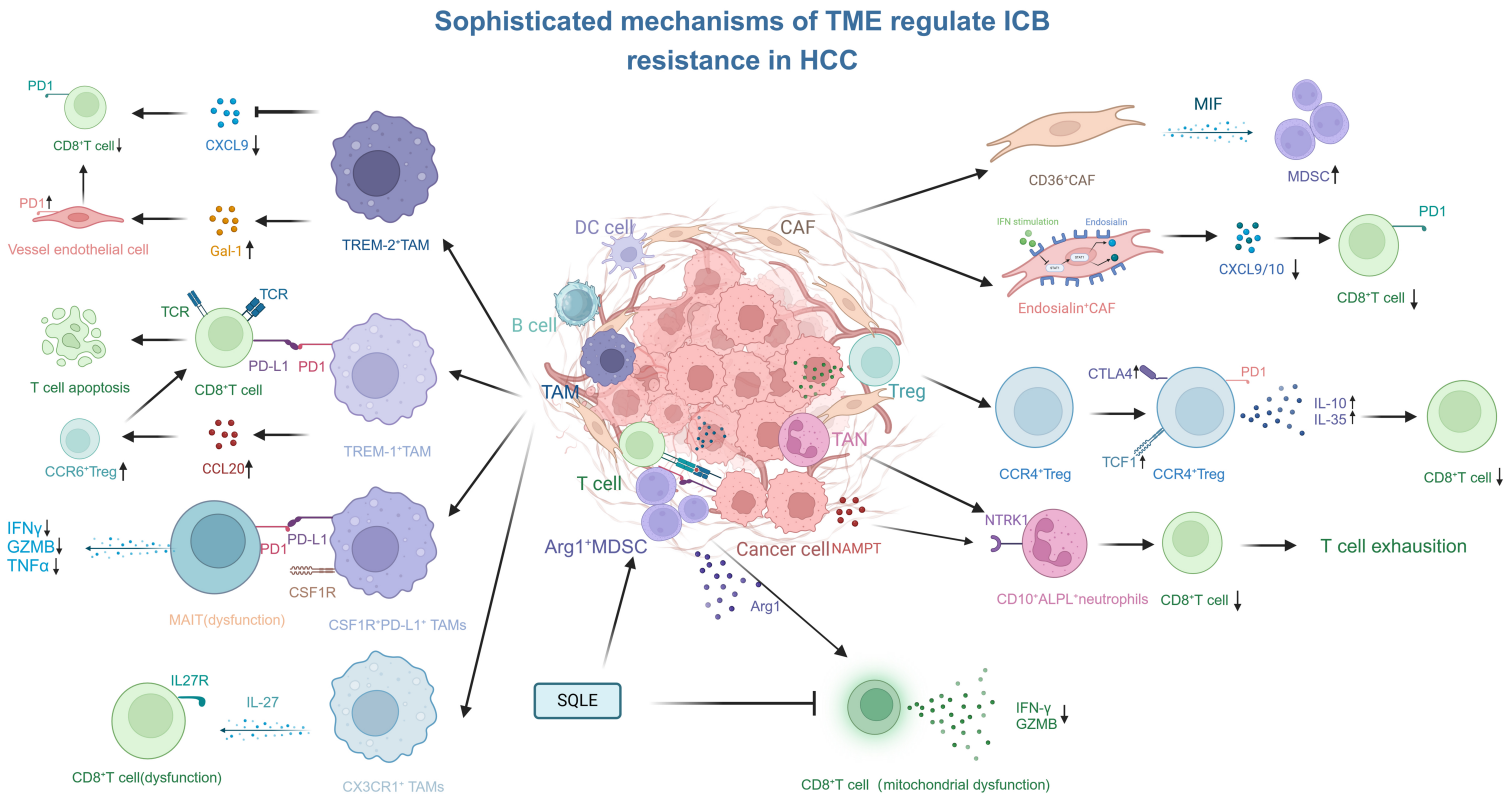
Sophisticated mechanisms of TME regulate ICB resistance in HCC. TME: Tumor microenvironment; ICB: immune-checkpoint blockade; HCC: hepatocellular carcinoma.

The major cell subtypes in the TME that regulate ICB resistance are summarized in Table 1. The underlying mechanisms of these subtypes are illustrated in the schematic diagram in Figure 1. Other cellular components, such as B cells and DCs, in the TME are also important for ICB response in HCC. However, the underlying mechanisms remain largely unknown and require further investigation.

**Table 1:** Mechanisms of various cell subtypes involved in ICB resistance of HCC

**Subtypes**	**Mechanisms of resistance**	**Strategies to enhance ICB**	**Ref.**
**CAFs and ICB response**			
POSTN^+^ CAFs	Recruit SPP1 + macrophages to construct immune barrier	Targeting POSTN^+^ CAFs and anti-PD-1	[24]
CD36^+^ CAFs	Release MIF to promote MDSC infiltration to establish immunosuppressive TME and induce cancer-stem property	CD36 inhibitor SSO^+^ anti-PD-1	[27]
Endosialin+ CAFs	Suppress STAT1-CXCL9/10 axis and reduce CD8^+^ T infiltration	Anti-endosialin and PD-1	[28]
FAP^+^ CAFs	Form a immune barrier with DAB2^+^ TAMs and block the infiltration of cytotoxic T cells into tumor	Combining FAP-CAR-T cell therapy and immune checkpoint inhibitors	[26]
**TAMs and ICB response**			
SPP1^+^ macrophage	Form a immune barrier with CAFs and block the infiltration of cytotoxic T cells into tumor	Anti-SPP1 and anti-PD-1	[23]
CX3CR1^+^ TAMs	Secret IL-27 to induce CD8^+^ T cell exhaustion and facilitate immune evasion	COX2 inhibitor celecoxib	[35]
Mac2^+^ macrophages	Form macrophages-coated tumor cluster and built immunosuppressive niche to trap cytotoxic T cells	Anti-PD-1 and GB1107	[36]
TREM-2^+^ TAMs	Reduce CXCL9 expression and augment the release of Gal-1	Anti-CSF1R and targeting Gal-1	[41,42]
TCR^+^ macrophages	High expression of cytotoxic related molecules and high expression of T cell-related genes	NA	[41]
TREM-1^+^ TAMs	Stimulate ERK/NF-κB signaling pathway	Blocking TREM-1 with specific inhibitor GF9 or anti-CCL20	[43]
CSF1R^+^ PD-L1^+^ TAMs	Contact with PD-1 of MAITs	Anti-PD1/PD-L1 therapy	[46]
Siglec-10hi TAMs	Express M2-like characteristics	Antibody Siglec-10 Fc treatment	[44,45]
DAB2^+^ TAMs	Form a immune barrier with FAP + CAFs and block the infiltration of cytotoxic T cells into tumor	Combining FAP-CAR-T cell therapy and immune checkpoint inhibitors	[26]
NOD1^+^ TAMs	Decrease expression of perilipin 5 to make favorable prognosis	NA	[47]
**TANs and ICB response**			
CD10^+^ ALPL^+^ neutrophils	Drive irreversible exhaustion of CD8^+^ T cells	Neutralizing antibodies against VEGFα or CXCL1	[52]
HLA-DR^+^ TANs	Trigger both (neo)antigen-specific and independent T cell responses	Neutrophil delivery or a leucine diet	[49]
PD-L1^+^ TANs	Reduce T cell infiltration, expression of cytotoxic GZMB and increased engagement of PD-1 and Tim3 on CD8^+^ T cells	Neutralizing antibodies against VEGFα or CXCL1	[52]
**MDSCs and ICB response**			
M-MDSCs	Diminish CD8^+^ T cells function and weaken immune surveillance	BET inhibitors	[61]
iNOS^+^ MDSCs	Secrete inhibitory factors that suppress T cell function	SQLE inhibitors terbinafine	[66]
Arg-1^+^ MDSC	Secreting Arg-1 to impair T cell function	Inhibition of SQLE (e.g., terbinafine) reduces MDSC immunosuppressive function	[66]
PMN-MDSCs	Weaken the anti-tumor effects of CD8^+^ T cells and NK cells	Anti-ly6g antibody	[68]
**Tregs and ICB response**			
CCR4^+^ Treg	Secreting immunosuppressive cytokines such as IL-10 and IL-35 inhibit the proliferation and function of CD8^+^ T cells	Using CCR4 antagonists such as C-021 or N-CCR4-Fc to block migration of CCR4^+^ Treg cells into the tumor microenvironment	[56]
CCR6^+^ Tregs	Interplay with TREM-1 + macrophages to induces T-cell apoptosis and dysfunction	Blocking TREM-1 with specific inhibitor GF9 or anti-CCL20	[43]
**Other cells and ICB response**			
MAITs	Recognize metabolites generated by the riboflavin synthesis pathway through its TCR and produce cytokines and cytotoxic molecules	NA	[46]
NKs	Mediate ADCC to enhance the effectiveness of ICB efficacy	NA	[73]
CD11b^+^ myeloid cells	Create an immunosuppressive microenvironment that limits CD8^+^ T cell activity	Combining CRA and anti-PD-1	[73]
IgA^+^ monocytes	Inhibit CD8^+^ T cell function directly	Combining FC-αr blocking peptides, anti-PD-L1 antibodies, and YAP/TAZ signaling pathway inhibitors	[81]
IgA^+^ macrophages	Inhibit CD8^+^ T cell function directly	Combining FC-αr blocking peptides, anti-PD-L1 antibodies, and YAP/TAZ signaling pathway inhibitors	[81]
LSECs	Form the specialized vascular bed structure	Combining simvastatin and anti-PD-L1	[100]

ICB: Immune checkpoint blockade; HCC: hepatocellular carcinoma; CAFs: cancer-associated fibroblasts; POSTN: periostin; SPP1: secreted phosphoprotein 1; MIF: macrophage migration inhibitory factor; MDSC: myeloid-derived suppressor cells; TME: tumor microenvironment; STAT1: signal transducer and activator of transcription 1; CXCL9/10: C-X-C motif chemokine ligand 9/10; FAP: fibroblast activation protein; DAB2: disabled homolog 2; TAMs: tumor-associated macrophages; CAR-T: chimeric antigen receptor T cell; COX2: cytochrome c oxidase subunit 2; TREM: triggering receptor expressed on myeloid cells; TCR: T cell receptor; ERK: extracellular signal-regulated kinase; NF-κB: nuclear factor kappa B; GF9: growth factor 9; PD-L1: programmed death-ligand 1; CCL20: C-C motif chemokine ligand 20; MAITs: mucosal-associated invariant T (MAIT) cells; NOD1: nucleotide binding oligomerization domain containing 1; TANs: Tumor-associated neutrophils; ALPL: alkaline phosphatase; VEGF: vascular endothelial growth factor; HLA-DR: human leukocyte antigen-DR; GZMB: granzyme B; MDSCs: myeloid-derived suppressor cells; BET: bromodomain and extra-terminal domain; iNOS: inducible nitric oxide synthase; SQLE: squalene epoxidase; PMN: polymorphonuclear; NK: natural killer cell; Tregs: regulatory T cell; CCR4: C-C motif chemokine receptor 4; ADCC: antibody-dependent cellular cytotoxicity; CRA: cryoablation; YAP: yes-associated protein; TAZ: transcriptional coactivator with PDZ-binding motif; LSECs: liver sinusoidal endothelial cells.

Notably, the TME is a complex ecosystem where each cellular component plays an interdependent role in regulating ICB response. There are intricate interplays among different cell types in the TME. For example, CAFs can capture TAMs, forming an immune barrier around the tumor to prevent CTL infiltration. Additionally, intratumoral CAFs can recruit MDSCs and Tregs to establish an immunosuppressive milieu that impedes CTL function. TAMs, in turn, can directly inhibit T cell infiltration and induce T cell exhaustion, facilitating immune evasion^[35]^. They can also activate ECs and attract Tregs, further suppressing CD8^+^ T cells. Besides the immune border formed by CAFs and TAMs, TAN-released NETs can encapsulate and shield tumor cells, protecting them from CD8^+^ T cell attack. Furthermore, the interaction between Tregs and DCs impairs antigen presentation and weakens T cell cytotoxicity. The coordinated actions of these various components in the TME jointly contribute to the resistance to ICB therapy in HCC.

At present, there remain some limitations in research on the TME and ICB response. First, most investigations just focus on specific cell types, such as CAFs, TAMs, and T cells, often overlooking the crosstalk of other cellular components in the TME. It is essential to explore the orchestrated mechanisms of the entire TME. Second, the TME is highly dynamic, evolving with the development of HCC and during ICB treatment. However, it is difficult to trace and monitor these dynamic changes in the TME throughout the therapeutic process in clinical settings. While some HCC patients are initially sensitive to ICB treatment, they may eventually develop acquired resistance. Understanding how critical TME characteristics transition from sensitivity to resistance remains an unresolved challenge. Comprehensive landscapes of the TME during ICB treatment need to be delineated. With the development of scRNA-seq and spatial transcriptomics, some specific cellular subtypes and distinctive spatial distributions of certain cellular communities associated with ICB response in HCC have been identified. Future studies should focus on elucidating the dynamic evolution, biological functions, and regulatory mechanisms of these subtypes and their spatial distribution. Last but not least, a significant gap exists between preliminary studies and clinical outcomes. The real-world conditions of HCC patients are much more complex than the widely used mouse models in research. Therefore, it is crucial to establish appropriate *in vivo* and *ex vivo* models that accurately reflect HCC development, ICB response, and resistance. Large-scale clinical trials are required to validate the accuracy and effectiveness of these preliminary findings.

## STRATEGIES TO IMPROVE ICB EFFICACY IN HCC

### Potential approaches to predict ICB efficacy

Imaging techniques, such as chemical-shift magnetic resonance imaging (MRI), can detect fat accumulation in HCC. Studies have shown that patients with higher tumor steatosis tend to respond better to ICB therapy. This suggests that imaging can serve as a tool to identify patients with high therapeutic potential who may benefit from ICB monotherapy or combined anti-PD-L1/anti-VEGF therapies^[19]^. Neoantigens, which are generated by tumor-specific mutations, can be recognized by the immune system. Patients with a high neoantigen burden are more likely to respond to ICB therapy. Predicting neoantigen load typically involves tumor genome sequencing and bioinformatics analysis^[102]^. Additionally, analysis of immune gene expression in the TME, such as genes related to the IFNγ pathway, helps identify patients likely to benefit from ICB. RNA sequencing (RNA-Seq) is commonly used for gene expression profiling (GEP) in this context^[102]^. The neutrophil-to-lymphocyte ratio (NLR) is a simple clinical marker that reflects the immune status of a patient. A high NLR suggests an immunosuppressive TME and is associated with poor responses to ICB, while a low NLR is typically observed in responders^[103-105]^. Specific gene expression patterns, such as high levels of IFNγ-related and antigen-presentation-related genes, are associated with favorable responses to immunotherapy. IFNγ-associated gene signatures (e.g., IFNAP signature) can help predict which patients are more likely to benefit from ICB^[15,103]^.

### Targeted therapy combined with immunotherapy

The combination of sorafenib and doxorubicin has demonstrated synergistic effects in controlling tumor progression, prolonging both progression-free survival (PFS) and OS in patients^[106]^. The COSMIC-312 trial is evaluating the efficacy of cabozantinib combined with atezolizumab versus sorafenib in advanced HCC. Although the trial is still ongoing, preliminary results indicate potential improvements in PFS and objective response rate (ORR) with the combination^[107]^. The combination of atezolizumab and bevacizumab has also shown remarkable clinical outcomes in HCC treatment. The IMbrave150 trial demonstrated that this combination significantly improved OS and PFS compared to sorafenib monotherapy. Bevacizumab enhances the immune-activating effects of atezolizumab by inhibiting angiogenesis and reducing immunosuppression within the TME^[10]^. In addition to the atezolizumab-bevacizumab combination, Sintilimab plus IBI305 (bevacizumab analog) has demonstrated superiority over sorafenib in the ORIENT-32 trial, particularly in the Asian patient population^[108]^. Other immune checkpoint inhibitors combined with targeted therapies are also being investigated. For instance, the combination of pembrolizumab and lenvatinib has shown promising clinical results^[109]^. The CARES-310 trial compared the efficacy of carrilizumab combined with ravoselanib and sorafenib as first-line treatment for advanced unresectable HCC. The results revealed that carrilizumab, in combination with ravoselanib, significantly extended PFS and OS compared with sorafenib^[110]^. Furthermore, neoadjuvant cabozantinib combined with nivolumab has shown a pathological response in 5 out of 15 HCC patients before surgical resection^[111]^.

### Multiple ICB combination therapy

The CheckMate 040 study led to the FDA’s accelerated approval of nivolumab and ipilimumab as a second-line therapy for advanced HCC. This combination regimen achieved an ORR of 33%, with over 30% of patients experiencing responses that lasted more than 24 months. However, the combination is associated with a high incidence of immune-related adverse events (irAEs), leading to treatment discontinuation in 18% of patients due to toxicity^[10,106]^. In the HIMALAYA trial, the combination of tremelimumab and durvalumab demonstrated significant efficacy, particularly in non-viral HCC patients, significantly improving OS (16.4 months *vs.* 13.8 months)^[112]^.

### Immunotherapy combined with locoregional therapy

Photothermal therapy (PTT) has been shown to reduce collagen content in the TME, enhancing immune cell infiltration and reducing Treg-mediated immunosuppression. When combined with ICB, PTT enhances CD8^+^ T cell function, significantly boosting antitumor immunity^[113]^. Furthermore, combining neoantigen vaccines with PD-1 inhibitors reduces Treg cell numbers and improves CD8^+^ T cell activity, thereby enhancing therapeutic outcomes^[114]^. Low-dose radiation therapy (LDRT), combined with dual VEGF and PD-L1 blockade, has been shown to activate intertumoral CD8^+^ T cells and improve immune responses, helping to overcome ICB resistance in HCC^[97]^. TACE, combined with PD-1 inhibitors such as pembrolizumab, has shown promise in boosting CD8^+^ T cell responses in HCC^[115]^. However, it should be noted that combined treatments such as TACE and ICB may lead to local resistance in the TME. For example, Tan *et al.* reported a significant increase in the number of TREM2^+^ TAMs following TACE. These TAMs release Gal-1, which decreases CXCL9 production and diminishes CD8^+^ T cell infiltration within the TME. Additionally, TREM2^+^ TAMs can upregulate PD-L1 expression on VECs, hindering T cell extravasation through the vascular wall into the tumor nest. These TREM2^+^ TAMs can also secrete VEGF-A and other factors that promote angiogenesis and support tumor growth. These changes may result from the low oxygen and low glucose conditions caused by TACE^[42]^. Herein, it is crucial to monitor the proportion of TREM2^+^ TAMs to prevent resistance when implementing combined TACE and ICB treatment.

We have listed the ongoing clinical trials of HCC immunotherapies in Table 2.

**Table 2:** Ongoing clinical trials of immunotherapy for liver cancer

**Trial (NCT number)**	**Population under study**	**Therapies under comparison**	**Primary end points**	**Sample size (*n*)**
NCT02562755	Patients with advanced hepatocellular carcinoma who have not received prior systemic therapy	Pexa-Vec followed by sorafenib *vs.* sorafenib	ORR	459
NCT03071094	Patients with advanced HCC	Immunotherapy Pexa-Vec with the PD-1 receptor blocking antibody nivolumab	ORR	14
NCT03575806	HCC patients with MVI after radical resection	TACE *vs.* TACE + TCM	RFS time	52
NCT00699816	Patients undergone curative resection (PEIT, RFA or operation) for HCC in Korea	CIK cell agent *vs.* no adjuvant treatment	RFS, RFS rate, OS	230
NCT02519348	Patients with advanced HCC	Durvalumab or tremelimumab monotherapy, or durvalumab in combination with tremelimumab or bevacizumab	DLTs, TEAEs and TESAEs, ORR, DCR, TTR, DoR, TTP, PFS, OS	433
NCT01853618	Patients with advanced liver cancer	Tremelimumab + RFA or TACE or cryoablation	Adverse event, best response, PFS, OS	61
NCT01147380	Liver transplant recipients with HCC	The dose of inoculation NK cells	Side effect, NK cell infusion-related toxicity	18
NCT03480152	People with metastatic melanoma or epithelial cancer	The dose of mRNA vaccine	Clinical response, adverse events, DLTs, MTD	5
NCT01967823	Patients with cancer that has the ESO-1 molecule on their tumors	Anti-ESO-1 cells	Clinical response, TCR in CD3 cell, related adverse events	11
NCT04099277	Patients with advanced solid tumors	LY3435151	DLTs, PK, Cmax, ORR, DCR, DoR, TTR, PFS	2
NCT02315066	Patients with select advanced or metastatic carcinoma	Dose of PF-04518600 *vs.* combine PF-05082566	DLTs, TEAEs and TESAEs	174

ORR: Overall response rate; HCC: hepatocellular carcinoma; MVI: microvascular invasion; TACE: transarterial chemoembolizatio; TCM: traditional Chinese medicine; RFS: recurrence-free survival; PEIT: percutaneous ethanol injection treatment; RFA: radiofrequency ablation; CIK: cytokine-induced killer; OS: overall survival; DLTs: dose limiting toxicities; TEAEs: treatment emergent adverse events; TESAEs: treatment emergent serious adverse events; DCR: disease control rate; TTR: time to response; DoR: duration of response; TTP: time to progression; PFS: progression free survival; NK: natural killer cell; MTD: maximum tolerated dose; TCR: T cell receptor; PK: pharmacokinetics; Cmax: maximum concentration.

The abbreviations are provided in Table 3.

**Table 3:** Abbreviation table

**Abbreviation**	**Full name**
HCC	Hepatocellular carcinoma
ICB	Immune checkpoint blockade
PD1	Programmed death 1
PD-L1	Programmed death-ligand 1
CTLA4	Cytotoxic T-lymphocyte-associatedprotein 4
HBV	Hepatitis B virus
HCV	Hepatitis C virus
VEGFR	Vascular endothelial growth factor receptor
FGFR	Fibroblast growth factor receptor
PDGFR	Platelet-derived growth factor receptor
TME	Tumor microenvironment
CAF	Cancer-associated fibroblast
TAM	Tumor-associated macrophage
TAN	Tumor-associated neutrophil
MDSC	Myeloid-derived suppressor cell
Treg	Regulatory T cell
NK	Natural killer cell
EC	Endothelial cell
EV	Extracellular vesicle
ECM	Extracellular matrix
scRNA-seq	Single-cell RNA sequencing
EMT	Epithelial-mesenchymal transition
IL-6	Interleukin 6
STAT3	Signal transducer and activator of transcription 3
SPP1	Secreted phosphoprotein 1
POSTN	Periostin
FOLR2	Folate receptor 2
PLVAP	Pigment epithelium-derived factor
FAP	Fibroblast activation protein
DAB2	Disabled homolog 2
TGF-β	Transforming frowth factor beta
PDGF	Platelet-derived growth factor
ADM	Adrenomedullin
OxLDL	Oxidized low-density lipoprotein
iNOS	Inducible nitric oxide synthase
IFN-γ	Interferon gamma
CXCL9	C-X-C Motif chemokine ligand 9
CXCL10	C-X-C Motif chemokine ligand 10
ZFP64	Zinc finger protein 64
CSF1	Colony stimulating factor 1
PKCα	Protein kinase C alpha
K63	Lysine 63
SOX18	SRY-box transcription factor 18
CXCL12	C-X-C motif chemokine ligand 12
CXCR4	C-X-C chemokine receptor type 4
ALK	Anaplastic lymphoma kinase
CacyBP	Calcyclin binding protein
Myd88	Myeloid differentiation primary response gene 88
CX3CL1	C-X3-C motif chemokine ligand 1
PEG2	Prostate-associated gene 2
CX3CR1	C-X3-C chemokine receptor type 1
COX2	Cytochrome c oxidase subunit 2
M2BP	Mac-2 binding protein
MCTC	Macrophages-coated tumor cluster
APOC1	Apolipoprotein C1
EVs	Extracellular vesicles
GOLM1	Golgi membrane protein 1
CSN5	COP9 signalosome 5
MISP	Mitotic spindle positioning
IQGAP1	IQ motif containing GTPase activating protein 1
siRNA	Small interfering RNA
TREM2	Triggering receptor expressed on myeloid cells 2
TCR	T cell receptor
TACE	Transarterial chemoembolizatio
Gal-1	Galectin 1
VECs	Vascular endothelial cells
HIF-1α	Hypoxia inducible factor 1 subunit alpha
TREM-1	Triggering receptor expressed on myeloid cells 1
CCL20	C-C motif chemokine ligand 20
CCR6	C-C motif chemokine receptor 6
MAIT	Mucosal-associated invariant T
NOD1	Nucleotide binding oligomerization domain containing 1
OX40L	TNF superfamily member 4
GSK3β	Glycogen synthase kinase 3 beta
ALPL	Alkaline phosphatase
NAMPT	Nicotinamide phosphoribosyltransferase
NTRK1	Neurotrophic receptor tyrosine kinase 1
CRKL	CRK like proto-oncogene adaptor protein
VEGF	Vascular endothelial growth factor
CXCL1	C-X-C motif chemokine ligand 1
GZMB	Granzyme B
NETs	Neutrophil extracellular traps
LRG1	Leucine rich alpha-2-glycoprotein 1
NASH	Nonalcoholic steatohepatitis
CXCR2	C-X-C motif chemokine receptor 2
Arg1	Arginase 1
HSCs	Hepatic stellate cells
PPARγ	Peroxisome proliferator-activated receptor gamma
VEGFA	Vascular endothelial growth factor A
LAPTM4B	Lysosomal protein transmembrane 4 beta
HMGB1	High mobility group box 1
MerTK	MER tyrosine kinase
SQLE	Squalene epoxidase
DCA	Dichloroacetate
IDO1	Indoleamine 2,3-dioxygenase 1
5-FU	5-fluorouracil
PMN-MDSCs	Polymorphonuclear MDSCs
iRFA	Incomplete radiofrequency ablation
cDC2	Conventional dendritic cell type 2
CXCL5	C-X-C motif chemokine ligand 5
GDF15	Growth differentiation factor 15
iTregs	Inducible Tregs
nTregs	Nature Tregs
STUB1	STIP1 homology and U-box containing protein 1
FOXP3	Forkhead box P3
SOX12	SRY-box transcription factor 12
CCL22	C-C motif chemokine ligand 22
CCR4	C-C motif chemokine receptor 4
ADCC	Antibody-dependent cellular cytotoxicity
UHRF1	Ubiquitin like with PHD and ring finger domains 1
TIM-3	Hepatitis A virus cellular receptor 2
ILC1	Type 1 innate lymphoid cell
CCAR1	Cell division cycle and apoptosis regulator 1
TP53	Tumor protein 53
mTORC1	CREB-regulated transcription coactivator
GSDMD	Gasdermin D
cGAS	Cyclic GMP-AMP synthase
SLAMF7	SLAM family member 7
CCL2	C-C motif chemokine ligand 2
CCR2	C-C motif chemokine receptor 2
MWA	Microwave ablation
CRA	Cryoablation
SRSF10	Serine and arginine rich splicing factor 10
MYB	MYB proto-oncogene
GLUT1	Glucose transporter 1
HK1	Hexokinase 1
LDHA	Lactate dehydrogenase A
GCDH	Glutaryl-CoA dehydrogenase
PGD	Phosphogluconate dehydrogenase
TKT	Transketolase
ALDOC	Aldolase C fructose-bisphosphate
CS	Cholesterol sulfate
DOCK2	Dedicator of cytokinesis 2
SULT2B1	Sulfotransferase family 2B member 1
NAFLD	TSC complex subunit 2
METTL3	Methyltransferase 3
INF-γ	Interferon gamma
VEGFR2	Vascular endothelial growth factor
CTNNB1	Catenin beta 1
MMP9	Matrix metallopeptidase 9
SSH1	Slingshot protein phosphatase 1
GPCR	G protein coupled recepto
FosB	FosB proto-oncogene
HIF	Transcription factor protein
FOXM1	Forkhead box M1
TPX4	Thioredoxin peroxidase 4
TPX2	Thioredoxin peroxidase 2
CXCR5	C-X-C motif chemokine receptor 5
FASN	Fatty acid synthase
SULT2B1	Sulfotransferase family 2B member 1
ZNF207	Zinc finger protein 207
PRMT2	Protein arginine methyltransferase 2
PRMT3	Protein arginine methyltransferase 3
mtDNA	Mitochondrial DNA
Ang2	Angiopoietin 2
EC	Endothelial cell
DGKG	Diacylglycerol kinase gamma
USP16	Ubiquitin specific peptidase 16
ZEB2	Zinc finger E-box binding homeobox 2
LSECs	Liver sinusoidal endothelial cells
NKT	Natural killer T cell
RNA-Seq	RNA sequencing
GEP	Gene expression profiling
NLR	Neutrophil-to-lymphocyte ratio
PFS	Progression-free survival
OS	Overall survival
ORR	Objective response rate
irAEs	Immune-related adverse events
PTT	Photothermal therapy
SF	Sorafenib
ICD	Immunogenic cell death
CRT	Calreticulin
CTLs	Cytotoxic T lymphocytes
APCs	Antigen-presenting cells
TLR	Toll-like receptor
LDRT	Low-dose radiation therapy
DPVB	Dual PD-L1/VEGF blockade
dLNs	Draining lymph nodes
CD8 Tef	CD8^+^ effector T cells
M1NVs	M1-type macrophages
OCA	Obeticholic acid
V-scVLPs	Virus-like silicon nanoparticles
GPC3	Glypican-3
CAR-T	Chimeric antigen receptor T cell
AGK	Acylglycerol kinase
CCL19	C-C motif chemokine ligand 19
NKG2D	Natural killer group 2 member D
VISTA	V-set immunoregulatory recepto
LAG-3	Lymphocyte activating 3
TIGIT	T cell immunoreceptor with Ig and ITIM domains

### Nanotechnology-based strategies combined with immunotherapy

Nanotechnology-based drug delivery systems have been developed to improve the penetration of drugs targeting CAFs in the TME. These nanoparticles can overcome stromal barriers created by CAFs, increasing the local concentration of drugs within tumors^[18,21,111]^. Micelles and liposomes, when modified with specific ligands, enable precise targeting of CAFs or tumor cells, minimizing systemic toxicity^[18]^. Nanovesicles derived from M1-type macrophages (M1NVs) have also shown efficacy in enhancing antitumor immunity^[116]^. Nanoparticles, such as a nano-PD-L1 trap, can decrease MDSC levels and increase CD8^+^ T cell infiltration, improving outcomes of ICB therapy^[117]^. A nanotransformation system that combines PD-L1 blockade with anti-angiogenic therapy has shown promise by reshaping the TME and reducing MDSC and Treg infiltration^[118]^. Additionally, obeticholic acid (OCA) delivered through a nanoemulsion system targets LSECs, recruiting NKT cells and enhancing antitumor immunity^[119]^. A magnetic nanocomposite system (HAPF) has been developed to label and activate NK cells, increasing their cytotoxicity under a magnetic field and improving ICB efficacy^[120]^. Another strategy involves a CRISPR/Cas9 nanodelivery system, which enhances NK cell-mediated tumor cell killing and improves HCC responses to ICB^[121]^. Nanotechnology-based therapies, such as virus-like silicon nanoparticles (V-scVLPs), have been used to deliver neoantigens to DCs, promoting CD8^+^ T cell activation and enhancing ICB efficacy^[122]^. A novel oxygen-catalyzing nanoplatform (BHMDI) has also been developed to increase CD8^+^ T cell infiltration and activity, thereby improving antitumor immune responses in combination with photodynamic therapy^[123]^.

### CAR-T combined with immunotherapy

Glypican-3 (GPC3) is one of the most extensively studied antigens in HCC. Preclinical studies have demonstrated that GPC3-targeted CAR-T cells effectively inhibit tumor growth^[10]^. However, in PD-L1-positive HCC models, the cytotoxic efficacy of GPC3-CAR-T cells is significantly reduced, though combining PD-1 inhibitors significantly enhanced their antitumor activities^[124]^. CAR-T cells overexpressing GLUT1 or AGK have demonstrated potent antitumor effects, suggesting that combining CAR-T therapy with ICB could further enhance the metabolic activity of CD8^+^ T cells and improve treatment outcomes in HCC^[125]^. Moreover, studies have shown that CAR-T cells engineered to co-secrete IL-7 and CCL19 (7 × 19 CAR-T cells) exhibit superior tumor suppression, proliferation capacity, and tumor infiltration in HCC xenograft models^[126]^. Makkouk *et al.* further improved CAR-T cell antitumor efficacy by combining them with Vδ1 γδ T cells. These engineered cells express a GPC3-specific CAR and secrete soluble IL-15 (sIL-15), promoting T cell expansion and survival, thereby augmenting antitumor responses^[127]^. Lu *et al.* developed a novel GPC3-IL7-CCL19-CAR-T cell that demonstrated significantly increased cytotoxicity against GPC3-positive HCC cells (e.g., HepG2), with a 1.5 to 2-fold higher killing efficiency compared to conventional GPC3-CAR-T cells^[128]^. Additionally, bispecific CAR-T cells targeting both GPC3 and PD-1 reduce the risk of tumor escape while maintaining prolonged cytotoxicity against PD-L1-positive HCC cells. AFP-CAR-T cells, which recognize AFP peptides bound to HLA complexes, have demonstrated potent antitumor effects in both in vitro and *in vivo* models. CD133 CAR-T cells have also shown promising antitumor activity in patients with advanced HCC. NKG2D CAR-T cells, which target NKG2D ligand-expressing HCC cells, exhibit strong cytotoxicity, and the use of non-viral methods to generate these cells has resulted in improved safety and efficacy^[129]^.

## CONCLUSION

Immunotherapy has brought new hope and a paradigm shift in the treatment of HCC. However, due to the complex microenvironment of the liver - particularly in the context of chronic liver disease - ICB therapy still faces significant challenges, such as low response rates, primary or acquired resistance, and potential systemic side effects. ICB resistance is driven by multiple factors, including T cell exclusion and dysfunction within the TME, defects in antigen processing, a lack of tumor-associated antigens, and the presence of alternative inhibitory immune checkpoints such as VISTA, TIM-3, LAG-3, and TIGIT. Additionally, external immunosuppressive factors - such as Tregs, TAMs, and inhibitory cytokines like TGF-β - contribute to the development of so-called “cold” tumors that are resistant to immunotherapy. This review summarizes the various mechanisms by which immune cells within the TME contribute to ICB resistance. The rapid advancement and widespread application of single-cell sequencing and spatial multi-omics technologies have significantly deepened our understanding of immune cell subtypes. However, many of these subtypes and their underlying mechanisms remain to be fully elucidated. Numerous ongoing research and clinical trials are exploring several promising strategies to enhance the efficacy of ICB treatment in HCC, including combinations of targeted therapies with immunotherapy, the use of multiple ICB agents, integration of ICB with other compounds, the application of nanotechnology, and the potential of CAR-T cell therapy. Future research efforts should focus on identifying optimal combination therapies, determining which HCC patients will benefit most from immunotherapies, and managing systemic side effects. Ultimately, deciphering the sophisticated mechanisms of ICB resistance is the essential cornerstone to improve the overall prognosis of HCC.

## DECLARATIONS

### Copyright

© The Author(s) 2025.

## References

[1] Sung H, Ferlay J, Siegel RL (2021). Global cancer statistics 2020: GLOBOCAN estimates of incidence and mortality worldwide for 36 cancers in 185 countries. CA Cancer J Clin.

[2] Kim YS, Shin SW (2019). Hepatocellular carcinoma. N Engl J Med.

[3] Li X, Ramadori P, Pfister D, Seehawer M, Zender L, Heikenwalder M (2021). The immunological and metabolic landscape in primary and metastatic liver cancer. Nat Rev Cancer.

[4] Yang C, Zhang H, Zhang L (2023). Evolving therapeutic landscape of advanced hepatocellular carcinoma. Nat Rev Gastroenterol Hepatol.

[5] Shi J, Liu J, Tu X (2022). Single-cell immune signature for detecting early-stage HCC and early assessing anti-PD-1 immunotherapy efficacy. J Immunother Cancer.

[7] Yau T, Kang YK, Kim TY (2020). Efficacy and safety of nivolumab plus ipilimumab in patients with advanced hepatocellular carcinoma previously treated with sorafenib: the CheckMate 040 randomized clinical trial. JAMA Oncol.

[8] Whiteside TL, Demaria S, Rodriguez-Ruiz ME, Zarour HM, Melero I (2016). Emerging opportunities and challenges in cancer immunotherapy. Clin Cancer Res.

[9] Chen C, Wang Z, Ding Y, Qin Y (2023). Tumor microenvironment-mediated immune evasion in hepatocellular carcinoma. Front Immunol.

[10] Oura K, Morishita A, Tani J, Masaki T (2021). Tumor immune microenvironment and immunosuppressive therapy in hepatocellular carcinoma: a review. Int J Mol Sci.

[11] Heymann F, Tacke F (2016). Immunology in the liver - from homeostasis to disease. Nat Rev Gastroenterol Hepatol.

[12] Fu T, Dai LJ, Wu SY (2021). Spatial architecture of the immune microenvironment orchestrates tumor immunity and therapeutic response. J Hematol Oncol.

[13] Wu Y, Cheng Y, Wang X, Fan J, Gao Q (2022). Spatial omics: navigating to the golden era of cancer research. Clin Transl Med.

[14] Li XY, Shen Y, Zhang L, Guo X, Wu J (2022). Understanding initiation and progression of hepatocellular carcinoma through single cell sequencing. Biochim Biophys Acta Rev Cancer.

[15] Yu L, Shen N, Shi Y (2022). Characterization of cancer-related fibroblasts (CAF) in hepatocellular carcinoma and construction of CAF-based risk signature based on single-cell RNA-seq and bulk RNA-seq data. Front Immunol.

[16] Woller N, Engelskircher SA, Wirth T, Wedemeyer H (2021). Prospects and challenges for T cell-based therapies of HCC. Cells.

[17] Mohr R, Jost-Brinkmann F, Özdirik B (2021). Lessons from immune checkpoint inhibitor trials in hepatocellular carcinoma. Front Immunol.

[18] Dong S, Guo X, Han F, He Z, Wang Y (2022). Emerging role of natural products in cancer immunotherapy. Acta Pharm Sin B.

[19] Murai H, Kodama T, Maesaka K (2023). Multiomics identifies the link between intratumor steatosis and the exhausted tumor immune microenvironment in hepatocellular carcinoma. Hepatology.

[20] Chen XQ, Ma J, Xu D, Xiang ZL (2023). Comprehensive analysis of KLF2 as a prognostic biomarker associated with fibrosis and immune infiltration in advanced hepatocellular carcinoma. BMC Bioinformatics.

[21] Llovet JM, Montal R, Sia D, Finn RS (2018). Molecular therapies and precision medicine for hepatocellular carcinoma. Nat Rev Clin Oncol.

[22] Liu Y, Ma J, Wang X (2023). Lipophagy-related gene RAB7A is involved in immune regulation and malignant progression in hepatocellular carcinoma. Comput Biol Med.

[23] Liu Y, Xun Z, Ma K (2023). Identification of a tumour immune barrier in the HCC microenvironment that determines the efficacy of immunotherapy. J Hepatol.

[24] Wang H, Liang Y, Liu Z (2024). POSTN^+^ cancer-associated fibroblasts determine the efficacy of immunotherapy in hepatocellular carcinoma. J Immunother Cancer.

[25] Li Z, Pai R, Gupta S (2024). Presence of onco-fetal neighborhoods in hepatocellular carcinoma is associated with relapse and response to immunotherapy. Nat Cancer.

[26] Long F, Zhong W, Zhao F (2024). *DAB2*
^+^ macrophages support *FAP*^+ ^fibroblasts in shaping tumor barrier and inducing poor clinical outcomes in liver cancer. Theranostics.

[27] Zhu GQ, Tang Z, Huang R (2023). CD36^+^ cancer-associated fibroblasts provide immunosuppressive microenvironment for hepatocellular carcinoma via secretion of macrophage migration inhibitory factor. Cell Discov.

[28] Gan L, Lu T, Lu Y (2024). Endosialin-positive CAFs promote hepatocellular carcinoma progression by suppressing CD8^+^ T cell infiltration. J Immunother Cancer.

[29] Xu G, Feng D, Yao Y (2020). Listeria-based hepatocellular carcinoma vaccine facilitates anti-PD-1 therapy by regulating macrophage polarization. Oncogene.

[30] Wang L, Hu YY, Zhao JL (2020). Targeted delivery of miR-99b reprograms tumor-associated macrophage phenotype leading to tumor regression. J Immunother Cancer.

[31] Wei CY, Zhu MX, Zhang PF (2022). PKCα/ZFP64/CSF1 axis resets the tumor microenvironment and fuels anti-PD1 resistance in hepatocellular carcinoma. J Hepatol.

[32] Chen J, Feng W, Sun M (2024). TGF-β1-induced SOX18 elevation promotes hepatocellular carcinoma progression and metastasis through transcriptionally upregulating PD-L1 and CXCL12. Gastroenterology.

[33] Liu C, Zhou C, Xia W (2024). Targeting ALK averts ribonuclease 1-induced immunosuppression and enhances antitumor immunity in hepatocellular carcinoma. Nat Commun.

[34] Wang J, Zhang X, Ma X (2023). Blockage of CacyBP inhibits macrophage recruitment and improves anti-PD-1 therapy in hepatocellular carcinoma. J Exp Clin Cancer Res.

[35] Xiang X, Wang K, Zhang H (2024). Blocking CX3CR1+ tumor-associated macrophages enhances the efficacy of anti-PD1 therapy in hepatocellular carcinoma. Cancer Immunol Res.

[36] Ning J, Ye Y, Shen H (2024). Macrophage-coated tumor cluster aggravates hepatoma invasion and immunotherapy resistance via generating local immune deprivation. Cell Rep Med.

[37] Hao X, Zheng Z, Liu H (2022). Inhibition of APOC1 promotes the transformation of M2 into M1 macrophages via the ferroptosis pathway and enhances anti-PD1 immunotherapy in hepatocellular carcinoma based on single-cell RNA sequencing. Redox Biol.

[38] Chen J, Lin Z, Liu L (2021). GOLM1 exacerbates CD8^+^ T cell suppression in hepatocellular carcinoma by promoting exosomal PD-L1 transport into tumor-associated macrophages. Signal Transduct Target Ther.

[39] Wang X, Ye X, Chen Y, Lin J (2023). Mechanism of M2 type macrophage-derived extracellular vesicles regulating PD-L1 expression via the MISP/IQGAP1 axis in hepatocellular carcinoma immunotherapy resistance. Int Immunopharmacol.

[40] Deng J, Ke H (2023). Overcoming the resistance of hepatocellular carcinoma to PD-1/PD-L1 inhibitor and the resultant immunosuppression by CD38 siRNA-loaded extracellular vesicles. Oncoimmunology.

[41] Li Y, Li F, Xu L (2024). Single cell analyses reveal the PD-1 blockade response-related immune features in hepatocellular carcinoma. Theranostics.

[42] Tan J, Fan W, Liu T (2023). TREM2^+^ macrophages suppress CD8^+^ T-cell infiltration after transarterial chemoembolisation in hepatocellular carcinoma. J Hepatol.

[43] Wu Q, Zhou W, Yin S (2019). Blocking triggering receptor expressed on myeloid cells-1-positive tumor-associated macrophages induced by hypoxia reverses immunosuppression and anti-programmed cell death ligand 1 resistance in liver cancer. Hepatology.

[44] Xiao N, Zhu X, Li K (2021). Blocking siglec-10^hi^ tumor-associated macrophages improves anti-tumor immunity and enhances immunotherapy for hepatocellular carcinoma. Exp Hematol Oncol.

[45] Li A, Ji B, Yang Y (2023). Single-cell RNA sequencing highlights the role of PVR/PVRL2 in the immunosuppressive tumour microenvironment in hepatocellular carcinoma. Front Immunol.

[46] Ruf B, Bruhns M, Babaei S (2023). Tumor-associated macrophages trigger MAIT cell dysfunction at the HCC invasive margin. Cell.

[47] Zhang F, Jiang Q, Cai J (2024). Activation of NOD1 on tumor-associated macrophages augments CD8^+^ T cell-mediated antitumor immunity in hepatocellular carcinoma. Sci Adv.

[48] Sun G, Liu H, Zhao J (2022). Macrophage GSK3β-deficiency inhibits the progression of hepatocellular carcinoma and enhances the sensitivity of anti-PD1 immunotherapy. J Immunother Cancer.

[49] Wu Y, Ma J, Yang X (2024). Neutrophil profiling illuminates anti-tumor antigen-presenting potency. Cell.

[50] Wang Y, Zhao Q, Zhao B (2022). Remodeling tumor-associated neutrophils to enhance dendritic cell-based HCC neoantigen nano-vaccine efficiency. Adv Sci.

[51] Meng Y, Ye F, Nie P (2023). Immunosuppressive CD10^+^ALPL^+^ neutrophils promote resistance to anti-PD-1 therapy in HCC by mediating irreversible exhaustion of T cells. J Hepatol.

[52] Xie P, Yu M, Zhang B (2024). CRKL dictates anti-PD-1 resistance by mediating tumor-associated neutrophil infiltration in hepatocellular carcinoma. J Hepatol.

[53] Yu Y, Zhang C, Dong B (2024). Neutrophil extracellular traps promote immune escape in hepatocellular carcinoma by up-regulating CD73 through Notch2. Cancer Lett.

[54] Zheng X, Yang L, Shen X (2024). Targeting *Gsk3a* reverses immune evasion to enhance immunotherapy in hepatocellular carcinoma. J Immunother Cancer.

[55] Leslie J, Mackey JBG, Jamieson T (2022). CXCR2 inhibition enables NASH-HCC immunotherapy. Gut.

[56] Gao Y, You M, Fu J (2022). Intratumoral stem-like CCR4+ regulatory T cells orchestrate the immunosuppressive microenvironment in HCC associated with hepatitis B. J Hepatol.

[57] Liang R, Hong W, Zhang Y (2023). Deep dissection of stemness-related hierarchies in hepatocellular carcinoma. J Transl Med.

[58] Kan A, Liu S, He M (2024). MZF1 promotes tumour progression and resistance to anti-PD-L1 antibody treatment in hepatocellular carcinoma. JHEP Rep.

[59] Xu N, Zhuo J, Chen Y (2024). Downregulation of N4-acetylcytidine modification in myeloid cells attenuates immunotherapy and exacerbates hepatocellular carcinoma progression. Br J Cancer.

[60] Wang L, Zhu L, Liang C (2023). Targeting N6-methyladenosine reader YTHDF1 with siRNA boosts antitumor immunity in NASH-HCC by inhibiting EZH2-IL-6 axis. J Hepatol.

[61] Liu M, Zhou J, Liu X (2020). Targeting monocyte-intrinsic enhancer reprogramming improves immunotherapy efficacy in hepatocellular carcinoma. Gut.

[62] Xiong Z, Chan SL, Zhou J (2023). Targeting PPAR-gamma counteracts tumour adaptation to immune-checkpoint blockade in hepatocellular carcinoma. Gut.

[63] Wang H, Zhou Q, Xie DF, Xu Q, Yang T, Wang W (2024). LAPTM4B-mediated hepatocellular carcinoma stem cell proliferation and MDSC migration: implications for HCC progression and sensitivity to PD-L1 monoclonal antibody therapy. Cell Death Dis.

[64] Conche C, Finkelmeier F, Pešić M (2023). Combining ferroptosis induction with MDSC blockade renders primary tumours and metastases in liver sensitive to immune checkpoint blockade. Gut.

[65] Wang S, Zhu L, Li T (2024). Disruption of MerTK increases the efficacy of checkpoint inhibitor by enhancing ferroptosis and immune response in hepatocellular carcinoma. Cell Rep Med.

[66] Wen J, Zhang X, Wong CC (2024). Targeting squalene epoxidase restores anti-PD-1 efficacy in metabolic dysfunction-associated steatohepatitis-induced hepatocellular carcinoma. Gut.

[67] Meng G, Li B, Chen A (2020). Targeting aerobic glycolysis by dichloroacetate improves Newcastle disease virus-mediated viro-immunotherapy in hepatocellular carcinoma. Br J Cancer.

[68] Kwong TT, Wong CH, Zhou JY (2021). Chemotherapy-induced recruitment of myeloid-derived suppressor cells abrogates efficacy of immune checkpoint blockade. JHEP Rep.

[69] Ren Y, Zhu L, Guo Y (2024). Melatonin enhances the efficacy of anti-PD-L1 by improving hypoxia in residual tumors after insufficient radiofrequency ablation. J Pharm Anal.

[70] Suthen S, Lim CJ, Nguyen PHD (2022). Hypoxia-driven immunosuppression by Treg and type-2 conventional dendritic cells in HCC. Hepatology.

[71] Wang Z, He L, Li W (2021). GDF15 induces immunosuppression via CD48 on regulatory T cells in hepatocellular carcinoma. J Immunother Cancer.

[72] Luo X, Huang W, Li S (2024). SOX12 facilitates hepatocellular carcinoma progression and metastasis through promoting regulatory T-cells infiltration and immunosuppression. Adv Sci.

[73] Tan J, Liu T, Fan W (2023). Anti-PD-L1 antibody enhances curative effect of cryoablation via antibody-dependent cell-mediated cytotoxicity mediating PD-L1^high^CD11b^+^ cells elimination in hepatocellular carcinoma. Acta Pharm Sin B.

[74] Zhang PF, Gao C, Huang XY (2020). Cancer cell-derived exosomal circUHRF1 induces natural killer cell exhaustion and may cause resistance to anti-PD1 therapy in hepatocellular carcinoma. Mol Cancer.

[75] Xiao R, Tian Y, Zhang J (2024). Increased Siglec-9/Siglec-9L interactions on NK cells predict poor HCC prognosis and present a targetable checkpoint for immunotherapy. J Hepatol.

[76] Heinrich B, Gertz EM, Schäffer AA (2022). The tumour microenvironment shapes innate lymphoid cells in patients with hepatocellular carcinoma. Gut.

[77] Hu Z, Chen G, Zhao Y (2023). Exosome-derived circCCAR1 promotes CD8 + T-cell dysfunction and anti-PD1 resistance in hepatocellular carcinoma. Mol Cancer.

[78] Yu J, Ling S, Hong J (2023). TP53/mTORC1-mediated bidirectional regulation of PD-L1 modulates immune evasion in hepatocellular carcinoma. J Immunother Cancer.

[79] Lv T, Xiong X, Yan W, Liu M, Xu H, He Q (2022). Targeting of GSDMD sensitizes HCC to anti-PD-1 by activating cGAS pathway and downregulating PD-L1 expression. J Immunother Cancer.

[80] Weng J, Wang Z, Hu Z (2024). Repolarization of immunosuppressive macrophages by targeting SLAMF7-regulated CCL2 signaling sensitizes hepatocellular carcinoma to immunotherapy. Cancer Res.

[81] Sung PS, Park DJ, Roh PR (2022). Intrahepatic inflammatory IgA^+^PD-L1^high^ monocytes in hepatocellular carcinoma development and immunotherapy. J Immunother Cancer.

[82] Cai J, Song L, Zhang F (2024). Targeting SRSF10 might inhibit M2 macrophage polarization and potentiate anti-PD-1 therapy in hepatocellular carcinoma. Cancer Commun.

[83] Lao Y, Cui X, Xu Z (2024). Glutaryl-CoA dehydrogenase suppresses tumor progression and shapes an anti-tumor microenvironment in hepatocellular carcinoma. J Hepatol.

[84] Wang S, Wang R, Xu N (2023). SULT2B1-CS-DOCK2 axis regulates effector T-cell exhaustion in HCC microenvironment. Hepatology.

[85] Seimiya T, Otsuka M, Fujishiro M (2023). Overcoming T-cell exhaustion: new therapeutic targets in HCC immunotherapy. Hepatology.

[86] Pan Y, Chen H, Zhang X (2023). METTL3 drives NAFLD-related hepatocellular carcinoma and is a therapeutic target for boosting immunotherapy. Cell Rep Med.

[87] Wang X, Zhou T, Chen X (2022). System analysis based on the cancer-immunity cycle identifies ZNF207 as a novel immunotherapy target for hepatocellular carcinoma. J Immunother Cancer.

[88] Pan B, Wang Z, Zhang X (2023). Targeted inhibition of RBPJ transcription complex alleviates the exhaustion of CD8^+^ T cells in hepatocellular carcinoma. Commun Biol.

[89] Wabitsch S, McCallen JD, Kamenyeva O (2022). Metformin treatment rescues CD8^+^ T-cell response to immune checkpoint inhibitor therapy in mice with NAFLD. J Hepatol.

[90] Cai N, Cheng K, Ma Y (2024). Targeting MMP9 in CTNNB1 mutant hepatocellular carcinoma restores CD8^+^ T cell-mediated antitumour immunity and improves anti-PD-1 efficacy. Gut.

[91] Hu B, Yu M, Ma X (2022). IFNα potentiates anti-PD-1 efficacy by remodeling glucose metabolism in the hepatocellular carcinoma microenvironment. Cancer Discov.

[92] Wang X, Wang J, Shen H, Luo Z, Lu X (2022). Downregulation of TPX2 impairs the antitumor activity of CD8+ T cells in hepatocellular carcinoma. Cell Death Dis.

[93] Huang J, Tsang WY, Fang XN (2024). FASN inhibition decreases MHC-I degradation and synergizes with PD-L1 checkpoint blockade in hepatocellular carcinoma. Cancer Res.

[94] Shi Y, Wu Z, Liu S (2024). Targeting PRMT3 impairs methylation and oligomerization of HSP60 to boost anti-tumor immunity by activating cGAS/STING signaling. Nat Commun.

[95] Song F, Hu B, Liang XL (2024). Anlotinib potentiates anti-PD1 immunotherapy via transferrin receptor-dependent CD8^+^ T-cell infiltration in hepatocellular carcinoma. Clin Transl Med.

[96] Dal Bo M, De Mattia E, Baboci L (2020). New insights into the pharmacological, immunological, and CAR-T-cell approaches in the treatment of hepatocellular carcinoma. Drug Resist Updat.

[97] Li S, Li K, Wang K (2023). Low-dose radiotherapy combined with dual PD-L1 and VEGFA blockade elicits antitumor response in hepatocellular carcinoma mediated by activated intratumoral CD8^+^ exhausted-like T cells. Nat Commun.

[98] Kikuchi H, Matsui A, Morita S (2022). Increased CD8+ T-cell infiltration and efficacy for multikinase inhibitors after PD-1 blockade in hepatocellular carcinoma. J Natl Cancer Inst.

[99] Ke J, Liu Y, Liu F (2024). In-situ-formed immunotherapeutic and hemostatic dual drug-loaded nanohydrogel for preventing postoperative recurrence of hepatocellular carcinoma. J Control Release.

[100] Yu Z, Guo J, Liu Y (2022). Nano delivery of simvastatin targets liver sinusoidal endothelial cells to remodel tumor microenvironment for hepatocellular carcinoma. J Nanobiotechnology.

[101] Zhang L, Xu J, Zhou S (2024). Endothelial DGKG promotes tumor angiogenesis and immune evasion in hepatocellular carcinoma. J Hepatol.

[102] Tang B, Zhu J, Zhao Z (2021). Diagnosis and prognosis models for hepatocellular carcinoma patient’s management based on tumor mutation burden. J Adv Res.

[103] Greten TF, Villanueva A, Korangy F (2023). Biomarkers for immunotherapy of hepatocellular carcinoma. Nat Rev Clin Oncol.

[104] Ao H, Xin Z, Jian Z (2021). Liquid biopsy to identify biomarkers for immunotherapy in hepatocellular carcinoma. Biomark Res.

[105] Hong JY, Cho HJ, Sa JK (2022). Hepatocellular carcinoma patients with high circulating cytotoxic T cells and intra-tumoral immune signature benefit from pembrolizumab: results from a single-arm phase 2 trial. Genome Med.

[106] Anwanwan D, Singh SK, Singh S, Saikam V, Singh R (2020). Challenges in liver cancer and possible treatment approaches. Biochim Biophys Acta Rev Cancer.

[107] Sperandio RC, Pestana RC, Miyamura BV, Kaseb AO (2022). Hepatocellular carcinoma immunotherapy. Annu Rev Med.

[108] Fulgenzi CAM, D’Alessio A, Airoldi C (2022). Comparative efficacy of novel combination strategies for unresectable hepatocellular carcinoma: a network metanalysis of phase III trials. Eur J Cancer.

[109] Llovet JM, Pinyol R, Kelley RK (2022). Molecular pathogenesis and systemic therapies for hepatocellular carcinoma. Nat Cancer.

[111] Zhang S, Yuan L, Danilova L (2023). Spatial transcriptomics analysis of neoadjuvant cabozantinib and nivolumab in advanced hepatocellular carcinoma identifies independent mechanisms of resistance and recurrence. Genome Med.

[112] Llovet JM, Willoughby CE, Singal AG (2023). Nonalcoholic steatohepatitis-related hepatocellular carcinoma: pathogenesis and treatment. Nat Rev Gastroenterol Hepatol.

[113] Yang H, Mu W, Yuan S (2024). Self-delivery photothermal-boosted-nanobike multi-overcoming immune escape by photothermal/chemical/immune synergistic therapy against HCC. J Nanobiotechnology.

[114] Yang SF, Weng MT, Liang JD (2023). Neoantigen vaccination augments antitumor effects of anti-PD-1 on mouse hepatocellular carcinoma. Cancer Lett.

[115] Zheng Z, Ma M, Han X (2023). Idarubicin-loaded biodegradable microspheres enhance sensitivity to anti-PD1 immunotherapy in transcatheter arterial chemoembolization of hepatocellular carcinoma. Acta Biomater.

[116] Chen Y, Li X, Shang H (2024). Mechanism exploration of synergistic photo-immunotherapy strategy based on a novel exosome-like nanosystem for remodeling the immune microenvironment of HCC. Nano Converg.

[117] Liu X, Zhou J, Wu H (2023). Fibrotic immune microenvironment remodeling mediates superior anti-tumor efficacy of a nano-PD-L1 trap in hepatocellular carcinoma. Mol Ther.

[118] Zhang Y, Zhong A, Min J (2024). Biomimetic responsive nanoconverters with immune checkpoint blockade plus antiangiogenesis for advanced hepatocellular carcinoma treatment. ACS Appl Mater Interfaces.

[119] Ji G, Ma L, Yao H (2020). Precise delivery of obeticholic acid via nanoapproach for triggering natural killer T cell-mediated liver cancer immunotherapy. Acta Pharm Sin B.

[120] Sim T, Choi B, Kwon SW (2021). Magneto-activation and magnetic resonance imaging of natural killer cells labeled with magnetic nanocomplexes for the treatment of solid tumors. ACS Nano.

[121] Zhang BC, Lai CM, Luo BY, Shao JW (2024). Triterpenoids-templated self-assembly nanosystem for biomimetic delivery of CRISPR/Cas9 based on the synergy of TLR-2 and ICB to enhance HCC immunotherapy. Acta Pharm Sin B.

[122] Lin Z, Jiang C, Wang P (2023). Caveolin-mediated cytosolic delivery of spike nanoparticle enhances antitumor immunity of neoantigen vaccine for hepatocellular carcinoma. Theranostics.

[123] Hou G, Qian J, Guo M (2022). Hydrazide-manganese coordinated multifunctional nanoplatform for potentiating immunotherapy in hepatocellular carcinoma. J Colloid Interface Sci.

[124] Carloni R, Sabbioni S, Rizzo A (2023). Immune-based combination therapies for advanced hepatocellular carcinoma. J Hepatocell Carcinoma.

[125] Sun RX, Liu YF, Sun YS (2024). GPC3-targeted CAR-T cells expressing GLUT1 or AGK exhibit enhanced antitumor activity against hepatocellular carcinoma. Acta Pharmacol Sin.

[126] Pang N, Shi J, Qin L (2021). IL-7 and CCL19-secreting CAR-T cell therapy for tumors with positive glypican-3 or mesothelin. J Hematol Oncol.

[127] Makkouk A, Yang XC, Barca T (2021). Off-the-shelf Vδ1 gamma delta T cells engineered with glypican-3 (GPC-3)-specific chimeric antigen receptor (CAR) and soluble IL-15 display robust antitumor efficacy against hepatocellular carcinoma. J Immunother Cancer.

[128] Lu LL, Xiao SX, Lin ZY (2023). GPC3-IL7-CCL19-CAR-T primes immune microenvironment reconstitution for hepatocellular carcinoma therapy. Cell Biol Toxicol.

[129] Aggeletopoulou I, Kalafateli M, Triantos C (2024). Chimeric antigen receptor T cell therapy for hepatocellular carcinoma: where do we stand?. Int J Mol Sci.

